# Transcriptional Responses in the Murine Spleen after *Toxoplasma gondii* Infection: Inflammasome and Mucus-Associated Genes

**DOI:** 10.3390/ijms18061245

**Published:** 2017-06-10

**Authors:** Eva B. Znalesniak, Ting Fu, Franz Salm, Ulrike Händel, Werner Hoffmann

**Affiliations:** 1 Institute of Molecular Biology and Medicinal Chemistry, Otto-von-Guericke University Magdeburg, 39120 Magdeburg, Germany; eva.znalesniak@med.ovgu.de (E.B.Z.); ftketty@gmail.com (T.F.); franz.salm@med.ovgu.de (F.S.); 2 Institute of Medical Microbiology and Hygiene, Otto-von-Guericke University Magdeburg, 39120 Magdeburg, Germany; ulrike.haendel@med.ovgu.de

**Keywords:** inflammation, inflammasome, TFF1, trefoil factor, *Toxoplasma gondii*, gastrokine, IgG Fc binding protein, MUC2

## Abstract

The spleen plays an important role in coordinating both adaptive and innate immune responses. Here, the transcriptional response to *T. gondii* infection in the murine spleen was characterized concerning inflammasome sensors (two different models: seven days after oral or four weeks after intraperitoneal infection). Additionally, Tff1^KO^ and Tff3^KO^ mice were investigated because *TFF* genes are often upregulated during inflammation. The expression of the pattern-recognition receptors Nlrp3, Nlrp12, and Nlrp1a was significantly increased after infection. This increase was diminished in Tff1^KO^ and Tff3^KO^ mice pointing towards a positive regulation of the inflammatory response by Tff1 and Tff3. Furthermore, the transcription of *Tff1* (encoding a motogenic lectin) and other secretory genes was analyzed, i.e., gastrokines (*Gkn*), IgG Fc binding protein (*Fcgbp*), and the mucin *Muc2*. The corresponding gene products belong to an interactome protecting mucous epithelia. Tff1 was significantly induced after infection, which might increase the motility of immune cells. In contrast, Gkn3, Fcgbp, and Muc2 were downregulated seven days after oral infection; whereas four weeks after i.p. infection only Gkn3 remained downregulated. This might be an indication that Gkn3, Fcgbp, and Muc2 are involved in the transient disruption of the splenic architecture and its reorganization, which is characteristic after *T. gondii* infection.

## 1. Introduction

The spleen is the largest secondary lymphoid organ of the body with various functions, the immune function being the most important one [1,2]. Here, phagocytosis, T cell-mediated immunity, and B cell-mediated humoral immunity occur mainly in the white pulp and the marginal zone of the spleen. The red pulp of the spleen is a major blood filter, is also involved in phagocytosis, and is a key site for iron metabolism; the latter being also a prerequisite particularly for the oxidizing function of monocytes.

The spleen is also a rich source for endocrine secretions, e.g., it is a key player in the “cytokine storm” that develops after infection and trauma [3,4]. For example, tumor necrosis factor (TNF)-α is produced in the spleen in high amounts and individuals having undergone splenectomy are highly susceptible to infections [5,6]. Of note, vagus nerve stimulation inhibits TNF-α production in the spleen at the transcriptional level by signaling through the α7 nicotinic acetylcholine receptor subunit [5]. Calcitonin gene-related peptide and β-endorphin are other endocrine peptides of the spleen [7,8]. Furthermore, the spleen also participates in metabolic and immunological abnormalities described in obesity, and splenectomy attenuates the progression of obesity and decreases insulin hypersecretion [9].

*Toxoplasma gondii* is a pathogen that infects all types of warm-blooded vertebrates. The parasite spreads by migration across biological barriers such as the intestine, the blood-brain barrier, the blood-retina barrier, and the placenta [10]. The host immune system plays a critical role in the response to *T. gondii* infection [11,12,13]. Immune factors involved in controlling *T. gondii* infection are, e.g., interleukin (IL)-6, IL-10, IL-12, IL-33, and interferon (IFN)-γ [4]. In humans, infections are normally subclinical and severe complications occur in immunocompromised patients and because of congenital infection. Recently, cytokine expression in the murine spleen has been investigated after intraperitoneal (i.p.) or oral *T. gondii* infection [4,14]. Of note, all the differentially expressed chemokines were upregulated; whereas most of the differentially expressed chemokine receptors were downregulated [4]. Furthermore, *T. gondii* infection caused a changed miRNA regulation network in mouse spleen as well as transcriptional changes of splenocyte organelle components [15,16].

In the past, we could show that i.p. *T. gondii* infection caused a significant induction of pattern-recognition receptors (PRRs) in the brain, particularly members of the NOD-like receptors and of the HIN200 family [17]. These intracellular sensors are, together with procaspase-1 and the adaptor protein ASC, typical constituents of inflammasomes [18,19,20]. Inflammasome activation leads to maturation of caspase-1 and the processing of the proinflammatory cytokines, IL-1β and IL-18. Thus, *T. gondii* effectors are master regulators of the inflammatory response and the inflammasome pathway [13]. However, there are no reports systematically analyzing the expression of inflammasome sensors in the spleen. Thus, we present here first data describing the expression of inflammasome sensors in the murine spleen in two different models of *T. gondii* infection, i.e., after oral (established ileitis model) or i.p. infection (established encephalitis model).

The two mouse models have been described in previous studies, where trefoil factor family 3 (Tff3)-deficient (Tff3^KO^) mice were also investigated after oral *T. gondii* infection (ileitis model) and Tff1^KO^ mice after i.p. *T. gondii* infection (encephalitis model), respectively [14,17]. In the present study, we continued our previous work and investigated the spleen of Tff3^KO^ mice after oral *T. gondii* infection because Tff3 is known to be expressed also in the spleen [14,21,22]. Furthermore, we investigated the spleen of Tff1^KO^ mice after i.p. *T. gondii* infection because Tff1 expression is known to be upregulated in the spleen after oral *T. gondii* infection [14]. Generally, TFF peptides (TFF1-3) are secretory lectins, which are expressed in mucous epithelia as well as the immune and the central nervous systems [21,23,24,25,26,27]. In the present study, other than inflammasome sensors, the splenic expression of *Tff1* and diverse secretory genes associated with Tffs, such as gastrokines (*Gkn*), IgG Fc binding protein (*Fcgbp*), and the mucin *Muc2*, was investigated. The corresponding gene products belong to an interactome protecting mucous epithelia. Particularly interesting is the expression of TFF1 because it has been shown to be typically upregulated during various chronic inflammatory processes [14,17].

## 2. Results

### 2.1. Expression Profiling of Mouse Spleen after Oral T. gondii Infection

**Figure 1:**
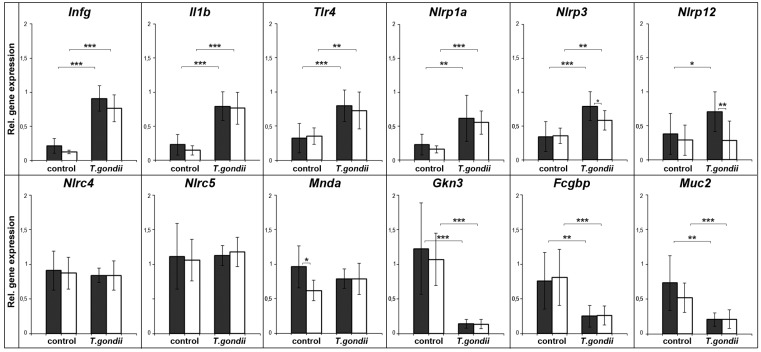
Semiquantitative RT-PCR analyses. *Ifng* (24×), *Il1b* (27×), *Tlr4* (31×), *Nlrp1a* (33×), *Nlrp3* (33×), *Nlrp12* (35×), *Nlrc4* (32×), *Nlrc5* (32×), *Mnda* (32×), *Gkn3* (35×), *Fcgbp* (35×), and Muc2 (35×) expression was monitored in the spleen seven days after oral *T. gondii* infection (ileitis model; 8 wild type and 11 Tff3^KO^ mice, respectively). As a control, the spleens of non-infected animals (nine wild type and nine Tff3^KO^ mice, respectively) were investigated. The relative gene expression levels were normalized against β-actin (*Actb*, 20×). The number of amplification cycles is given in parentheses. Significances are indicated by asterisks (*, *p* ≤ 0.05; **, *p* ≤ 0.01; ***, *p* ≤ 0.001). Wild type animals: black bars; Tff3^KO^ animals: white bars.

The expression of typical inflammatory marker genes was analyzed (validated by semi-quantitative evaluation) in wild type and in Tff3^KO^ animals seven days after oral *T. gondii* infection (Figure 1). To monitor the inflammatory process, signature genes such as interferon γ (*Ifnγ*), *Il1β*, and *Tlr4* were selected. As expected, these genes were significantly upregulated after *T. gondii* infection. The expression analysis of transcripts encoding the inflammasome constituents Nlrp1a, Nlrp3, Nlrp12, Nlrc4, Nlrc5, and Mnda revealed that Nlrp1a, Nlrp3, and Nlrp12 were significantly upregulated in *T. gondii* infected animals. Of note, Nlrp12 was only upregulated in wild type animals, but not in Tff3^KO^ mice. In contrast, the expression of the inflammasome sensors—Nlrc4, Nlrc5, and Mnda—was not changed significantly after *T. gondii* infection.

Furthermore, the expression of genes associated with TFF peptides and mucous epithelia—such as *Gkn3*, *Fcgbp*, and *Muc2*—was analyzed. These three genes were significantly downregulated after *T. gondii* infection.

### 2.2. Expression Profiling of Mouse Spleen after Intraperitoneal T. gondii Infection

**Figure 2:**
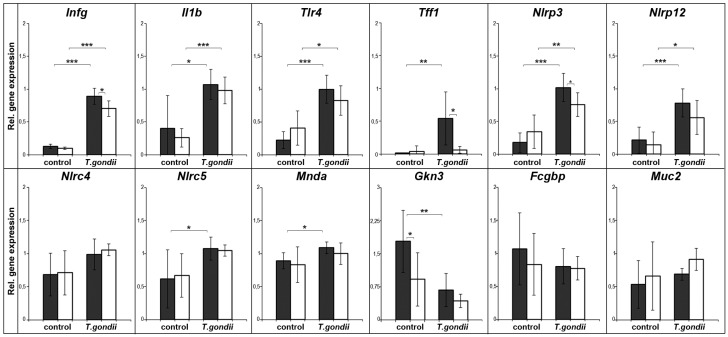
Semiquantitative RT-PCR analyses. *Ifng* (30×), *Il1b* (27×), *Tlr4* (32×), *Tff1* (32×), *Nlrp3* (33×), *Nlrp12* (35×), Nlrc4 (33×), Nlrc5 (33×), Mnda (33×), *Gkn3* (35×), *Fcgbp* (35×), and *Muc2* (35×) expression was monitored in the spleen four weeks after i.p. *T. gondii* infection (encephalitis model; six wild type and six Tff1^KO^ mice, respectively). As a control, the spleen of non-infected animals (six wild type and six Tff1^KO^ mice, respectively) was investigated. The relative gene expression levels were normalized against β-actin (*Actb*, 22×). The number of amplification cycles is given in parentheses. Significances are indicated by asterisks (*, *p* ≤ 0.05; **, *p* ≤ 0.01; ***, *p* ≤ 0.001). Wild type animals: black bars; Tff1^KO^ animals: white bars.

The expression of a similar set of genes was also analyzed in wild type and Tff1^KO^ animals four weeks after intraperitoneal *T. gondii* infection (Figure 2). Again, the inflammatory markers Ifnγ, Il1β, and Tlr4 were significantly upregulated in the infected animals. Furthermore, Tff1 was significantly upregulated after *T. gondii* infection as well as expression of the inflammasome constituents Nlrp3 and Nlrp12; whereas the expression of the inflammasome sensor Nlrc4 did not show a significant change compared to the moderate upregulation of Nlrc5 and Mnda. In contrast to Fcgbp and Muc2, only Gkn3 was significantly downregulated in infected animals.

**Figure 3:**
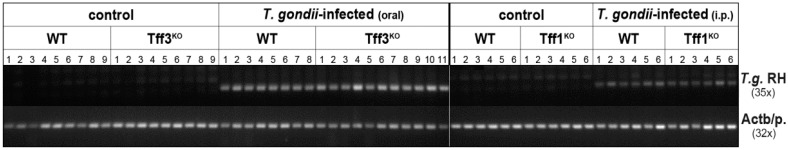
PCR analyses of genomic DNA from the spleen for the *T. gondii* RH strain repeat region (T.g. RH). *T. gondii* DNA was monitored seven days after oral *T. gondii* infection and four weeks after i.p. infection, respectively. As a control, DNA from the β-actin promoter (Actb/p.) was amplified. The number of amplification cycles is given in parentheses.

In order to confirm infection of the animals with *T. gondii*, the presence of the RH repeat region of *T. gondii* was monitored in the spleen in both infection models (Figure 3). Clearly, only the infected animals contained this DNA.

## 3. Discussion

### 3.1. T. gondii Infection Induces the Expression of Specific Inflammasomes in the Spleen

In both experimental models, Ifnγ, Il1β, and Tlr4 were upregulated in the spleen (Figure 1 and Figure 2). This is in line with previous reports describing increased splenic expression of these genes after *T. gondii* infection [4,14,28,29,30]. In human monocytes, particularly the secreted GRA15 protein of *T. gondii* is responsible for IL-1β induction and the release of IL-1β is a direct consequence of inflammasome activation after infection [31]. Thus, these genes serve as positive controls indicating inflammatory processes in the spleen after *T. gondii* infection in our experimental studies presented here. Furthermore, also expression of the *T. gondii* RH repeat region confirms the infection of the spleen (Figure 3).

Expression of the inflammasome sensors Nlrp3 and Nlrp12 is significantly increased (*p* ≤ 0.001) in both models of *T. gondii* infection (wild type animals). Of note, the upregulation of Nlrp3 and Nlrp12 in infected Tff3^KO^ and Tff1^KO^ animals is reduced when compared to the corresponding wild type animals. This might be an indication that Tff1 and Tff3 positively regulate the inflammatory process. This view is supported by the observation that Tff3^KO^ mice showed a reduced immune response in the ileum after oral *T. gondii* infection [14].

Furthermore, also Nlrp1a expression was significantly upregulated (*p* ≤ 0.001) seven days after oral *T. gondii* infection (animals with mixed 129/Sv and C57BL/6 background; Figure 1). In contrast, Nlrp1a expression was not detectable in the strains used for i.p. infection (129/Sv background). This is in line with previous reports that Nlrp1a and Nlrp1c expression is lacking in certain 129S1 mouse strains [17,32].

In contrast, Nlrc4 expression (and Nlrc5 and Mnda seven days after *T. gondii* infection) was not changed. Similar results were obtained for Nlrp6 and Aim2 (data not shown). Generally, the picture emerges that PRR expression after *T. gondii* infection is rather moderate, slow, and focal in the spleen, predominantly affecting Nlrp3 and Nlrp12. This is in contrast to the expression pattern in the brain four weeks after i.p. *T. gondii* infection, where at least Nlrp3, Nlrc4, Nlrc5, and Mnda were strongly upregulated (*p* ≤ 0.001) [17].

### 3.2. Splenic Tff1 Expression is Induced in Two Models of T. gondii Infection

Tff1 expression in the spleen was significantly induced (*p* ≤ 0.01) four weeks after i.p. *T. gondii* infection (Figure 2). This result is in agreement with a previous study showing significantly induced Tff1 expression in the spleen also seven days after oral *T. gondii* infection [14]. Thus, Tff1 is ectopically expressed in the inflamed spleen in two different models after *T. gondii* infection.

Taken together, this result is in line with ectopic TFF1 expression during various inflammatory processes, such as in the brain in an encephalitis model [17], in a murine ileitis model [14], during chronic intestinal ulceration [33], chronic pancreatitis [34], in the colon of infants with inflammatory bowel disease [35], in the porcine colon after infection with *Salmonella typhimurium* [36], and in a murine asthma model [37,38].

The upregulated Tff1 expression in the spleen after *T. gondii* infection is correlated with a complex inflammatory process. A primary response of the spleen is obviously the formation of inflammasomes (particularly Nlrp3 and Nlrp12) as shown in Figure 1 and Figure 2. Then, the release of IL1β and IL18 probably triggers NF-κB-dependent transcriptional events [19]. Induction of Tff1 expression as a consequence of TNF-α and IL1β stimulation and activation of NF-κB has been documented [39,40]. The specific upregulation of Tff1, but not of Tff2 and Tff3, could have been brought forth via FoxA (formerly: hepatocyte nuclear factor 3) and binding to motif IV [17,41,42,43]. Of note, FoxA expression is upregulated particularly by inflammatory cytokines [44].

Finally, the question arises concerning the biological function of ectopic TFF1 expression during inflammatory processes. TFF1 has been reported to have protective and healing effects to mucous epithelia and acts as a motogen (for reviews, see [21,23,24]); furthermore, it has a pH-dependent lectin activity [45]. Thus, Tff1 could influence, for example, the motility of certain immune cells. The reduced inflammatory response in Tff1^KO^ animals (especially concerning the expression of Ifnγ and Nlrp3; Figure 2) after i.p. *T. gondii* infection points towards a positive regulation of the inflammatory response by Tff1 in order to protect the organ against invasion of pathogens. Of note, a similar effect has been observed in Tff3^KO^ animals after oral *T. gondii* infection (particularly concerning Nlrp3, Nlrp12; Figure 1).

### 3.3. Changes of Other Secretory Genes in the Spleen after T. gondii Infection

Trefoil factor family (TFF) peptides are typical constituents of mucous gels and are also secreted from the central nervous system as well as the immune system [21,23,24,25,26,27]. In the spleen, particularly TFF2 and TFF3 are expressed [14,22,46,47]. The biosynthesis of TFF1 and TFF3 is complex; both are secretory peptides containing an odd number of cysteine residues and are able to form disulfide-linked heterodimers with GKN2 and FCGBP, respectively [48,49,50]. Thus, in the studies presented here the expression of secretory gastrokines, Fcgbp, and the gel-forming mucin Muc2—which are typically co-expressed in mucous epithelia—were monitored at the transcriptional level.

Surprisingly, Gkn3, Fcgbp, and Muc2 were significantly downregulated (*p* ≤ 0.001) seven days after oral *T. gondii* infection (Figure 1); whereas four weeks after i.p. *T. gondii* infection, only Gkn3 was downregulated (*p* ≤ 0.01). In contrast, the expression of Gkn1 and Gkn2 was hardly detectable in non-infected mice and rather increased little for Gkn2 after infection, particularly in Tff1^KO^ mice (data not shown). Thus, the expression of Gkn3, Fcgbp, and Muc2 is contrary to that of inflammatory genes. Furthermore, it seems that their downregulation is a rather transient and quick response, because the effect is most prominent in the acute infection and seems to be attenuated in chronic infection after four weeks (only Gkn3 is still significantly downregulated; however, one has to consider that different *T. gondii* strains were used in the two experimental models). Currently, the function of these genes in the spleen is not known and this is the first description of their transcription in this organ. Gkn3 has been reported to inhibit gastric epithelial cell proliferation and probably marks a distinct neck cell precursor population [51]. Thus, Gkn3 could reduce the number of cell divisions, which is known to be very low in the spleen [52]. Of note, GKN3 function has been lost in humans [51]. Fcgbp is an IgG Fc binding protein, which is entirely different from Fcγ receptors, and is able to attach covalently to the mucin Muc2 [53,54]. Of special note, it has been postulated that Fcgbp traps HIV-1-antibody complexes at mucosal surfaces [55]. Thus, Fcgbp and Muc2 would be perfectly designed to establish an extracellular matrix with a barrier or adhesive function, particularly for immunoglobulins. Such a molecular function would be in agreement with the physiological role of the spleen. Furthermore, infection with *T. gondii* is known to induce a transient disruption of the splenic architecture [56]. As a consequence, the transiently reduced expression of Gkn3, Fcgbp, and Muc2 after *T. gondii* infection could well be a sign, that these genes are involved in the splenic reorganization.

Furthermore, the expression of ependymin related protein 1 (Epdr1, previously termed Merp2) was monitored, because it was downregulated in a murine asthma model [37]. Epdr1 probably encodes a lysosomal protein [57] homologous to human UCC1/MERP1 [58]. In both models, Epdr1 transcript levels did not significantly change after *T. gondii* infection (data not shown). This is comparable to a constant cerebral Epdr1 expression after i.p. *T. gondii* infection [17].

Analysis of the cellular localization of Tff1, Gkn3, Fcgbp, and Muc2 might be an interesting topic for further studies in order to gain more insights into the molecular functions of these new players in splenic function.

## 4. Materials and Methods

### 4.1. Murine T. gondii Infection Models

Two infection models described in detail previously [14,17] were applied for the studies presented here. First, corresponding wild type and Tff3^KO^ animals (mixed 129/Sv and C57BL/6 background), respectively were orally infected with three cysts of a type II strain (ME49) per mouse (ileitis model) and seven days post-infection the spleen was collected as described [14]. Second, corresponding wild type and Tff1^KO^ animals (129/Sv background) were i.p. infected with five cysts of the type II DX strain per mouse (encephalitis model) and, four weeks post-infection, the spleen was collected as reported [17]. Procedures concerning animal care and the generation of data from animal samples were according to legal regulations; *T. gondii* infection studies were approved by the responsible state authorities (No. 42502-2-1233 UniMD, 01/2014 and 12/2016; No. 42502-2-1004 UniMD, 09/2010, 11/2013, 03/2015; Landesverwaltungsamt Sachsen-Anhalt, Halle, Germany).

### 4.2. DNA and RNA Extraction, PCR Analysis

Genotyping the different mouse strains from tail clippings was as previously described [14,17]. Infection of the spleen with *T. gondii* was monitored by amplifying the *T. gondii* RH strain repeat region from 150 ng genomic DNA from the spleen. The specific primer pairs used have been published previously (*Actb*/promoter, MB1783/MB1784; [14]) or are listed in Table 1 (RH repeat region, MB2066/MB2067).

The isolation of total RNA of murine tissues as well as RT-PCR analysis and semi-quantitative evaluation of relative gene expression levels including statistical analysis have already been described in detail [14,17]. The specific primer pairs used in this RT-PCR study have been also published previously (*Actb*, MB1912/MB1913; *Ifnγ*, MB2054/MB2055; *Il1β*, MB2038/MB2039; *Tff1*, MD7/MD8; *Nlrp1a*, MB2576/MB2577; *Nlrp3*, MB2584/MB2585; *Nlrc4*, MB2382/MB2383; *Nlrc5*, MB2608/MB2609; *Mnda*, MB2600/MB2601; [14,17]) or are listed in Table 1 (*Fcgbp*, *Gkn3*, *Muc2*, *Nlrp12*, Toll-like receptor/*Tlr4*).

## 5. Conclusions

In two different models of *T. gondii* infection (oral and i.p., respectively), the splenic expression of specific inflammasome sensor genes (*Nlrp3*, *Nlrp12*) was upregulated together with typical inflammatory marker genes (*Ifng*, *Il1b*, *Tlr4*). Of note, the inflammatory response was diminished in Tff1^KO^ and Tff3^KO^ mice, which points towards a pro-inflammatory role of Tff1 and Tff3. Furthermore, *Tff1* expression was also significantly upregulated after *T. gondii* infection. This established again *Tff1* as a marker gene for inflammatory processes. In contrast, the splenic expression of certain mucus-associated genes (*Gkn3*, *Fcgbp*, *Muc2*) was downregulated particularly seven days after oral *T. gondii* infection. This might be a sign that these genes are involved in the transient disruption of the splenic architecture and its reorganization after *T. gondii* infection.

## Figures and Tables

**Table 1:** Oligonucleotides used for (RT)-PCR analysis and calculated size of the products.

Genes	Accession No.	Primer No.	Primer Pairs	Nucleotide Positions	*T*m	Size (bp)	Intron Spanning
*Fcgbp*	NM_001122603.1	MB1516	CCAAAACCTGGAGATGAGGA	6215–6234	60 °C	621	Yes
		MB1517	CAGGCTACGGCAGAGATAGG	6835–6816			
*Gkn3*	NM_026860.1	MB2656 MB2657	TGGTCAGCATCCGAGACAAC CATGAGTCTGGGTCCATCGT	270–289 612–593	60 °C	343	Yes
*Muc2*	NM_023566.3	MB2660 MB2661	GCTCTTTCTTCCTACGCCCG CATGAAGGTATGGTCAGGGC	1913–1933 2141–2122	60 °C	228	Yes
*Nlrp12*	NM_001033431.1	MB2606 MB2607	CCCGTTACTTTGTCCCCCAT CACGCTGATTGGCTCTCAAAA	184–203 536–516	60 °C	353	Yes
*Tlr4*	NM_021297.3	MB1687	AGAAAATGCCAGGATGATGC	269–288	60 °C	417	Yes
		MB1688	GTCTCCACAGCCACCAGATT	685–666			
*T. g.* RH repeat region	AF487550.1	MB2066 MB2067	ACTACAGACGCGATGCCGCTC CTCTCCGCCATCACCACGAGGAA	107–127 328–306	60 °C	222	
